# Subliminal Instrumental Conditioning Demonstrated in the Human Brain

**DOI:** 10.1016/j.neuron.2008.07.005

**Published:** 2008-08-28

**Authors:** Mathias Pessiglione, Predrag Petrovic, Jean Daunizeau, Stefano Palminteri, Raymond J. Dolan, Chris D. Frith

**Affiliations:** 1 Wellcome Trust Centre for NeuroImaging, Institute of Neurology, University College London, 12 Queen Square, London WC1N 3BG, UK; 2 Laboratoire INSERM U610, Centre de NeuroImagerie de Recherche (CENIR), Institut Fédératif de Recherche en Neurosciences, Hôpital Pitié-Salpêtrière, Université Pierre et Marie Curie (Paris 6), 47 Boulevard de l'Hôpital 75013 Paris, France

**Keywords:** SYSNEURO

## Abstract

How the brain uses success and failure to optimize future decisions is a long-standing question in neuroscience. One computational solution involves updating the values of context-action associations in proportion to a reward prediction error. Previous evidence suggests that such computations are expressed in the striatum and, as they are cognitively impenetrable, represent an unconscious learning mechanism. Here, we formally test this by studying instrumental conditioning in a situation where we masked contextual cues, such that they were not consciously perceived. Behavioral data showed that subjects nonetheless developed a significant propensity to choose cues associated with monetary rewards relative to punishments. Functional neuroimaging revealed that during conditioning cue values and prediction errors, generated from a computational model, both correlated with activity in ventral striatum. We conclude that, even without conscious processing of contextual cues, our brain can learn their reward value and use them to provide a bias on decision making.

## Introduction

Humans frequently invoke an argument that their intuition can result in a better decision than conscious reasoning. Such assertions may rely on subconscious associative learning between subliminal signals present in a given situation and choice outcomes. For instance, clinicians may improve their therapeutic decisions through learned associations between treatment outcomes and subliminal signs presented by their patients. Likewise, poker players can improve their gambles through a learned association between monetary outcomes and subliminal behavioral manifestations of their opponents (the so-called “gamblers' tell”).

The idea that such instrumental conditioning can occur subconsciously has been around for almost a century (Thorndike, 1911). This assumption originally rested on observations that rewards and punishments shape behavioral responses in species allegedly lacking conscious awareness. However, subliminal conditioning studies in humans have so far been restricted to Pavlovian paradigms such as fear conditioning (Clark and Squire, 1998; Knight et al., 2003; Morris et al., 1998; Olsson and Phelps, 2004), where subliminal stimuli (like unseen faces) are paired with unpleasant events (like white noise) to increase automatic responses (like skin conductance). To our knowledge, subliminal instrumental conditioning, where decision making would be biased by unperceived cues predicting rewards or punishments, has never been previously demonstrated.

Our subliminal conditioning task was adapted from a published supraliminal task, wherein subjects selected between visual cues so as to learn choices that maximized monetary outcomes (Pessiglione et al., 2006). In our previous study, we modeled subjects' behavior by optimizing the free parameters of a standard machine learning algorithm (termed Q learning), to get maximal likelihoods for the observed decisions. When we regressed key output variables of the optimized model against simultaneously acquired functional neuroimaging data, we showed that prediction errors were expressed in the striatum. Postexperimental debriefing indicated that some subjects managed to understand the statistical structure of the task, while others appeared to rely on what they referred to as their intuition. These latter reports suggest that subjects can improve their decisions without consciously following the incremental steps of the Q-learning procedure.

The motivating assumption of the current experiment was that processes associated with striatal learning are not consciously accessible but, nonetheless, influence choice decision making. Indeed, if contextual cues reach awareness, other brain systems are likely to play a role, as expressed in conscious reasoning or strategic control, which allows one to develop explicit knowledge of statistical contingencies. However, if the cues remain unseen, learning would solely depend on a subconscious processing that involves the striatum, with an algorithmic structure similar to a Q learning, which does not embody explicit information about statistical contingencies. Under these assumptions, we predicted that, if in our task visual cues were masked, both striatal activity and behavioral choices would still reflect Q-learning outputs.

## Results

A prerequisite for the present study was to demonstrate efficient masking of the visual cues. These cues were novel abstract symbols, which were scrambled and mixed to create mask images. To assess visual awareness, we successively displayed two masked cues on a computer screen and asked subjects whether they perceived a difference or not. We reasoned that if subjects are unable to correctly perceive any difference between the masked cues, then they are also unable to build conscious representations of cue-outcome associations. The procedure has the advantage of not showing the cues unmasked, so that, by the end of the experiment, subjects had no idea what the cues look like.

The perceptual discrimination task was performed outside the scanner at the beginning of the experiment, in order to adapt duration of cue display to each individual, and in the scanner at the end of the experiment, to check for any effect of learning or change in visual conditions. For all subjects, cue duration was set at either 33 or 50 ms and was kept fixed through the entire experiment. In every individual, correct guessing on the final assessment did not differ from chance (p > 0.05, chi-square test). At group level, average percentage of correct responses for the 20 subjects was 48% ± 3%, which again was not different from chance (p > 0.5, two-tailed paired t test). Average d′ was 0.08 ± 0.20, showing that, even when correcting for response bias, signal detection was not different from zero (p > 0.5, two-tailed paired t test). Thus, subjects remained unable to discriminate between the different masked cues, from the beginning to the end of conditioning sessions.

**Figure 1:**
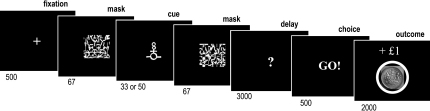
Subliminal Conditioning Task Successive screenshots displayed during a given trial are shown from left to right, with durations in milliseconds. After seeing a masked contextual cue flashed on a computer screen, subjects choose to press or not press a response button and subsequently observe the outcome. In this example, “Go” appears on the screen because the subject has pressed the button, following the cue associated with a rewarding outcome (winning £1).

We employed the same masking procedure in the subliminal conditioning task, in which cues were paired with monetary outcomes (Figure 1). From these outcomes (−£1, £0, +£1), subjects had to learn when to take the risky response (either “Go” or “NoGo,” depending on subjects). Subjects were also told that, for the risky response, the outcome would depend on the cue hidden behind the masking image (see instructions in Supplemental Data available online). As they would not see the cues, we encouraged them to follow their intuition, taking the risky response if they had a feeling they were in a winning trial and choosing a safe response if they felt it was a losing trial. Note that if subjects always made the same response, or if they performed at chance, their final payoff would be zero.

**Figure 2:**
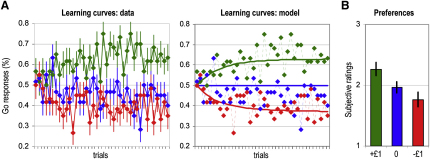
Behavioral Data (A) Learning curves. Colors indicate cues for which button presses are rewarded (green), neutral (blue), or punished (red). Diamonds represent, across trials, percentages of subjects that pressed the button. Left: continuous lines join the diamonds to illustrate actual choices made by subjects. Right: continuous lines represent the probabilities of button press estimated by an optimized Q-learning model. (B) Preferences. After the conditioning phase, cues were unmasked and subjects rated them, from the most (3) to the least liked (1). The graph shows the average rating for reward (green), neutral (blue), and punishment (red) cues. Bars are ± intersubjects standard errors of the mean.

As a dependent variable to assess for conditioning effects, we used monetary payoff, which corresponds to the area below the reward and above the punishment learning curves (Figure 2A). Overall subjects won money in this task, on average £7.5 ± £1.8 (p < 0.001, one-tailed paired t test), indicating that the risky response was more frequently chosen following reward predictive relative to punishment predictive cues. Both reward and punishment conditions also differed significantly from the neutral condition (p < 0.05, one-tailed paired t test). There was no significant difference (p > 0.5, two-tailed paired t test) between the reward and punishment condition: on average subjects won £24.3 ± £1.9 and avoid losing £23.2 ± £2.1. Learning curves showed that responses improved symmetrically for rewards and punishments, ending with 63% ± 5% of correct responses on average. Surprisingly, this plateau was reached at around the halfway point of the learning session. The effects of subliminal conditioning were subsequently assessed with a preference judgment task, in which cues were uncovered and rated by the subjects from the most to least liked (Figure 2B). Ratings were significantly higher for reward compared to punishment cues (p < 0.01, one-tailed paired t test), consistent with subjects having learned the affective values of subliminal cues, such that these values were able to bias their preferences. When uncovering the cues, subjects were also asked to signal any cue that they may have seen during conditioning sessions; none was reported as previously seen.

To model instrumental conditioning, we implemented a standard Q-learning algorithm (Pessiglione et al., 2006), with inputs from individual histories of cues, choices, and outcomes. On every trial, the model estimates the likelihood of the risky response from the value of the displayed cue. If the risky response was actually taken, the model then updates the value of the displayed cue in proportion to the prediction error. The parameters of the model were optimized such that likelihoods of risky responses provided the best fit of subjects' actual responses across conditioning sessions (Figure 2A). The Q values and prediction errors generated by this optimized algorithm were then used as regressors for analysis of brain imaging data (see Figure S1).

**Figure 3:**
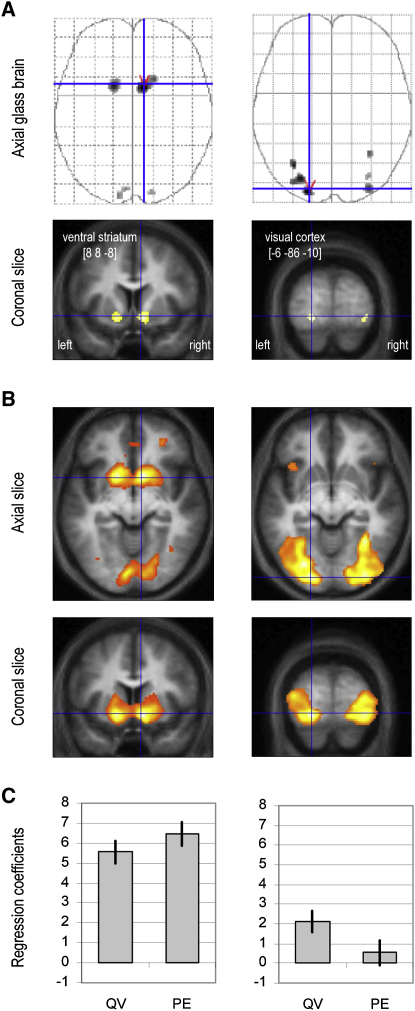
Neuroimaging Data Left: ventral striatal regions isolated by regression of monetary values against BOLD responses to cue display. Right: visual cortical regions isolated by correlation of cue-value regression coefficients with individual payoffs. Slices were taken at global maxima of interest indicated by red pointers on the above axial glass brains. Areas shown in gray/black on glass brains and in orange/yellow on coronal slices showed significant effect. The [x y z] coordinates of the maxima refer to the Montreal Neurological Institute space. (A) Statistical parametric maps using conservative threshold (p < 0.05 after familywise error correction for multiple comparisons). (B) Statistical parametric maps using liberal threshold (p < 0.001 uncorrected). (C) Regression coefficients of Q values (QV) and prediction errors (PE) against BOLD responses to cue and outcome display, respectively. Bars are ± intersubjects standard errors of the mean.

**Figure 4:**
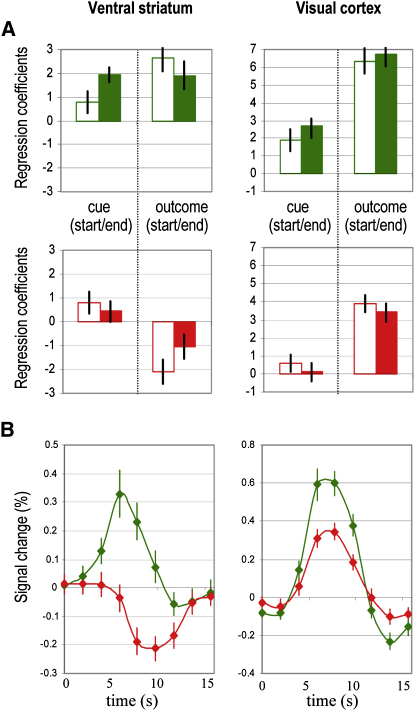
Model-free Analyses of Brain Activations Ventral striatum (left) and visual cortex (right) correspond to voxels surviving familywise error correction on statistical parametric maps. (A) Regression coefficients. Histograms represent contrasts of the reward (green) or the punishment (red) condition with the neutral condition, at the times of cue and outcome display. For every contrast the two joint histograms correspond to the first (empty) and second (filled) halves of the conditioning sessions. (B) Time courses. BOLD responses were averaged across trials over conditioning sessions, for the reward (green) and punishment (red) conditions, relative to the neutral condition. Bars are ± intersubjects standard errors of the mean.

We recorded brain activity while subjects performed the subliminal conditioning task, using functional magnetic resonance imaging (fMRI). We first examined brain regions reflecting Q value at the time of cue onset, increasing their response to reward-predicting cues and decreasing their response to punishment-predicting cues, across learning sessions. After correction for multiple comparisons (family-wise error, p < 0.05), we noted significant correlated activity in ventral striatum bilaterally (Figures 3A and 3B, left). The same region was also significantly activated at the time of outcome in keeping with prediction errors being expressed at this time point (Figure 3C, left). In a second analysis, we computed regression coefficients for the different conditions at the time of cue and outcome onsets, separately for the first and second half of conditioning sessions. Contrasts with the neutral condition were then averaged over all ventral striatal voxels showing significant activation at the most conservative threshold in the first analysis. This confirmed that from the first to the second half of conditioning sessions, ventral striatal responses increased for reward cues and decreased for punishment cues (Figure 4A, left). At the time of outcome onset, the same ventral striatal region reflected positive prediction errors in the reward condition and negative prediction errors in the punishment condition. In keeping with the Q-learning model, both positive and negative prediction errors decreased from the first to the second half of conditioning sessions. Thus, across subliminal conditioning, the ventral striatal response was consistent with the expression of Q values (for unseen cues) and prediction errors (based on visible outcomes).

We further examined variability in individual performance to explain why some subjects won more money than others. More precisely, we searched for brain regions where coefficients of Q-value regressors correlated with individual payoffs. These regions were confined to extrastriate visual cortex (Figure 3A, right) at the most conservative threshold (familywise error, p < 0.05), spreading into the ventral occipitotemporal stream (Figure 3B, right) with a more liberal threshold (uncorrected, p < 0.001). Contrast estimates confirmed that extrastriate voxels progressively differentiated the reward and punishment cues from the first to the second half of conditioning sessions (Figure 4A, right). At the time of outcome onset, these extrastriate regions responded positively for both rewards and punishments, showing no evidence for encoding of prediction errors. Thus, during the subliminal conditioning task, the extrastriate visual cortex learned to discriminate between unseen cues according to their reward value but did not express outcome-related prediction errors (Figure 3C, right).

To further assess whether the ventral striatum and visual cortex were able to discriminate between the subliminal cues, we extracted time courses of BOLD response. These time courses were averaged over trials, sessions, and subjects, separately for the reward and punishment conditions (Figure 4B). We found that BOLD responses to reward and punishment cues significantly differed after two acquisition volumes (3.9 s) in the ventral striatum (one-tailed paired t test, p < 0.01) and after three (5.85 s) in the visual cortex (one-tailed paired t test, p < 0.01).

Finally, we ascertained whether neuroimaging and behavioral effects of subliminal conditioning were driven by subjects scoring at the high end in perceptual discrimination performance. We tested for correlations between correct guessing assessed in the final perceptual discrimination test and coefficients of Q-value regressors in both the ventral and extrastriate cortex. None was significant; Pearson's correlation coefficients were respectively −0.25 and −0.18. We also tested correlation of correct guessing with monetary payoffs from conditioning sessions and differential ratings in the preference judgment task. Again, none was significant; Pearson's correlation coefficients were respectively 0.26 and 0.29.

## Discussion

We provide evidence that instrumental learning can occur without conscious processing of contextual cues. This finding might relate to previous evidence for implicit or procedural learning, where behavioral responses can be adapted to the statistical structure of stimuli that fails to be reported explicitly (Bayley et al., 2005; Berns et al., 1997; Destrebecqz and Cleeremans, 2001; Seitz and Watanabe, 2003). Interestingly, implicit/procedural learning has been suggested to involve the basal ganglia, in contrast with explicit/declarative memory which would involve the medial temporal lobe (Cohen et al., 1985; Milner et al., 1998; Poldrack and Packard, 2003; Squire, 1992). In implicit learning tasks, such as serial reaction time or probabilistic classification, authors have claimed that subjects can achieve good acquisition without explicit knowledge of the task structure. However, methods for assessing awareness of statistical contingencies have been criticized, principally on the issue that questions were too demanding in terms of memory (Lagnado et al., 2006; Lovibond and Shanks, 2002; Wilkinson and Shanks, 2004). Thus, to formally test whether instrumental conditioning can occur without awareness, we took a more stringent approach: masking the cues, so that they remained unperceived.

We believe our methodology avoids most previous problems related to assessing awareness, by demonstrating that subjects were not able to discriminate between masked cues (without the help of rewards and punishments), rather than retrospectively assessing awareness of contingencies. Moreover, postconditioning recognition tests would not be sufficient in our case, since subjects would not need to identify cues for associative learning to be conscious. Indeed, they could learn associations between risky response outcomes and tiny fragments of the visual dynamic pattern formed by the mask/cue/mask flashing. However, postconditioning debriefing questions might be informative in explaining why subjects could not discriminate between masked cues. Thus, when we explicitly asked subjects to state what the cues looked like, they reported in majority of cases that they had no idea. When the subjects were presented with the cues, now unmasked, they reported surprise at seeing the symbols while asserting that they had never seen them before. This suggests that during conditioning, subjects had no a priori representational knowledge to guide a visual search for cues hidden behind the masks. We believe that absence of an a priori representation is a crucial feature of our design, which, in addition to visual masking, prevented subjects from consciously seeing the cues.

Using this methodology, we show that pairing rewards and punishments can guide behavioral responses and even condition preferences for abstract cues that subjects could not consciously see. Note that if cues were visible, learning curves would have been optimized in one trial or two; hence we are not claiming that conscious awareness is unhelpful in supraliminal instrumental conditioning. However, in our subliminal conditioning task, conscious strategies (such as win-stay/lose-switch) might have been detrimental, which would explain why learning curves were limited well below the optimum.

We also identified brain circuitry associated with subliminal instrumental conditioning. The ventral striatum responded to subliminal cues and to visible outcomes in a manner that closely approximates Q-learning algorithm, expressing reward expected values and prediction errors, just as was reported in supraliminal instrumental conditioning studies (O'Doherty et al., 2004; Pagnoni et al., 2002; Pessiglione et al., 2006; Yacubian et al., 2006). Interestingly, there is no need for representing the statistical structure of the task in Q learning, which is an incremental procedure updating the expected values of chosen actions according to the subject's history of reward and punishment outcomes. This accords well with views that the striatum is a major player in implicit/procedural learning (Graybiel, 2005; Hikosaka et al., 1999; Packard and Knowlton, 2002) and with evidence that ventral striatum encodes reward-related information (Delgado, 2007; Knutson and Cooper, 2005; Pecina et al., 2006).

For the sake of simplicity, we have described ventral striatum activity as directly reflecting key outputs of Q-learning algorithm: Q value at the time of cue onset and prediction error at the time of outcome. There are nonetheless other variables in machine learning literature that would also correlate with ventral striatum activity and which could provide an alternative interpretational framework for our study. In particular, it is important to note that average Q values (over the reward, neutral, and punishment conditions) remain around zero during our conditioning paradigm. Hence, Q value is approximately equal to Q value minus average Q value, which can be seen as equivalent to a cue prediction error (actual Q value minus predicted Q value). Our data are therefore equally compatible with the notion that the ventral striatum encodes prediction errors at the time of both cue and outcome onsets. However, because prediction errors represent a function of Q values, the brain has to learn about Q values in order to signal prediction errors. Thus, whether we consider the ventral striatum as encoding a Q value or a prediction error does not alter our central conclusion: namely, the human brain can learn the reward value of subliminal cues, so as to later influence behavioral choices.

It is of interest that extrastriate visual cortex also reflected the reward value of subliminal cues, but not outcome-related prediction errors. Modulation of visual cortex activity by monetary incentives has already been reported in neuroimaging studies of supraliminal processes, such as visuomotor transformation, attentional control, and working memory (Krawczyk et al., 2007; Ramnani and Miall, 2003; Small et al., 2005). In our case, the modulation suggests that conditioning involves an interaction between the extrastriate cortex (which would discriminate cues according to their visual properties) and the ventral striatum (which would tag cues with affective values depending on reward prediction errors). However, we acknowledge that we do not as yet have a complete account of how the brain produces behavioral effects of subliminal conditioning. Notably, we failed to identify the brain regions mapping affective values onto motor commands, which would complete the circuit from visual cues to behavioral responses. Further experiments will be necessary to fill in these explanatory gaps.

More generally, our approach, combining perceptual masking and computational modeling, can be extended over the field of functional neuroimaging. Computational reinforcement learning theory has proven useful to model both brain activity and behavioral choices in human and nonhuman primates (Daw and Doya, 2006; McClure et al., 2004; O'Doherty et al., 2007). Brain activity reflecting sophisticated computations are unlikely to be accessed by the conscious mind, which takes minutes to solve even simple equations. This brain activity would therefore represent unconscious processes, which we formally demonstrated here in the case of instrumental conditioning. Combining masking and modeling can, in principle, make more tractable the identification of basic neuronal mechanisms shared within other species, eliminating the use of reportable knowledge that might be unique to humans. It might also help assess the integrity of these same basic mechanisms in patients with neurological or psychiatric conditions, avoiding confounds generated by conscious strategic compensations.

## Experimental Procedures

### Subjects

The study was approved by the National Hospital for Neurology and Neurosurgery and the Institute of Neurology joint Ethics Committee. Subjects were recruited via Gumtree website and screened for exclusion criteria: left handedness, age below 18 or above 39, regular taking of drug or medication, history of psychiatric or neurological illnesses and contra-indications to MRI scanning (pregnancy, claustrophobia, metallic implants). All subjects gave informed consent prior to taking part. We scanned 20 subjects: 11 males (mean age 26.8 ± 6.3 years) and 9 females (mean age 23.8 ± 3.3 years). Two more subjects were scanned but discarded from the analysis, because they eventually could describe parts of the visual cues, were above chance level in the perception task, and won unusually large amounts of money in the conditioning task. Subjects were told that they would play for real money, but at the end of the experiment their winnings were rounded up to a fixed amount.

### Behavioral Task and Analysis

Subjects first read the instructions (see Supplemental Data) about the different tasks, which were later explained again step by step. Before scanning, subjects were trained to perform the conditioning task and the perception task on practice versions. In the scanner, they had to perform three sessions of the conditioning task, each containing 120 trials and lasting 13 min, and one session of the perception task, containing 120 trials and lasting about 7 min. The abstract cues were letters taken from the Agathodaimon font. The 12 cues shown in the scanner were randomly assigned to the four task sessions, each session hence employing 3 new cues. The same two masking patterns (see Figure 1), one displayed before and the other after the cue, were used in all task sessions. The sequence of display and the cue-outcome associations were also randomized for every subject.

The perceptual discrimination task was used to select the appropriate duration for cue display, which was then kept to either 33 or 50 ms for the entire experiment. In this task, subjects were flashed two masked cues, 3 s apart, displayed on the center of a computer screen, each following a fixation cross. They had to report whether or not they perceived any difference between the two visual stimulations. The response was given manually, by pressing one of two buttons assigned to “same” and “different” choices. The perceptual discrimination task was then employed as a control for awareness at the end of conditioning sessions. We checked with a chi-square test that in all included subjects performance was not significantly different from chance level (50% of correct responses). We also calculated d′ measure, which is the difference between normalized rates of hits (correct “different” response) and false alarms (incorrect “different” responses). We ensured that this measure was not significantly different from zero, at group level, using one-tailed paired t test.

The instrumental conditioning task involved choosing between pressing or not pressing a button, in response to masked cues. After showing the fixation cross and the masked cue, the response interval was indicated on the computer screen by a question mark. The interval was fixed to 3 s and the response was taken at the end: “Go” if the button was being pressed, “No” if the button was released. The response was written on the screen as soon as the delay had elapsed. Subjects were told that one response was safe (you do not win or lose anything) while the other was risky (you can win £1, lose £1, or get nothing). The risky response was assigned to Go for half of the subjects, and to NoGo for the other half, such that motor aspects were counterbalanced between reward and punishment conditions. Subjects were also told that the outcome of the risky response would depend on the cue that was displayed between the mask images. In fact, three cues were used, one was rewarding (+£1), one was punishing (−£1), and the last was neutral (£0). Because subjects were not informed about the associations, they could only learn them by observing the outcome, which was displayed at the end of the trial. This was either a circled coin image (meaning +£1), a barred coin image (meaning −£1), or a gray square (meaning £0).

Subjects were then debriefed about their visual perceptions and their response strategies. They reported responding either at chance, following their intuition, or following logical rules. None of them had the slightest idea of what the cues looked like. For the preference judgment task, the cues were then shown unmasked on a computer screen. The three cues used for a given session were displayed side by side, the position being randomized. Subjects were asked to rate them in order of preferences: 3 for the most liked, 2 for the intermediate, and 1 for the least one.

To assess for instrumental conditioning, we used one-tailed paired t tests comparing individual earnings with chance level (which is £0). Similarly, to assess for preference conditioning, we used one-tailed paired t tests comparing differential rating of winning and losing cues with chance level (which is 0).

### Computational Model

We used a standard Q-learning algorithm (Sutton and Barto, 1998), which has been shown previously to offer a good account of instrumental choice in both humans and monkeys (Daw and Doya, 2006; McClure et al., 2004; O'Doherty et al., 2007). For each cue, the model estimates the expected value of the risky response, on the basis of individual sequences of choices and outcomes. This value, termed a Q value, is essentially the amount of reward expected from choosing the risky response given the contextual cue. These Q values were set at 0.1 before learning, and after every risky response the value of the cue was updated according to the Rescorla-Wagner rule: Q(t + 1) = Q(t) + α^∗^δ(t). Following this rule, values are increased if the outcome is better than expected, and decreased in the opposite case. The prediction error was δ(t) = R(t) − Q(t), with R(t) defined as the reinforcement obtained from choosing the risky response at trial t. In other words, the prediction error δ(t) is the difference between the expected outcome, i.e., Q(t), and the actual outcome, i.e., R(t). The reinforcement magnitude R was +1 and −1 for winning and losing £1, and 0 for neutral outcomes. Given the Q value, the associated probability of choosing the risky response was estimated by implementing the softmax rule: P(t) = 1/(1 + exp(−Q(t)/β)). This rule ensures that likelihood will be superior to 0.5 for positive values and inferior to 0.5 for negative values. The learning rate α concerns the amplitude of value changes from one trial to the next. The temperature β concerns the randomness of decision making. These two free parameters, α and β, were adjusted to maximize the probability (or likelihood) of the actual choices under the model. With the constraint that the parameters should be identical for reward and punishment cues we found: α = 0.1 and β = 0.9. The model was then used to create statistical regressors corresponding to the Q values and prediction errors, for analysis of brain images.

### Images Acquisition and Analysis

T2^∗^-weighted echo planar images (EPI) were acquired with blood oxygen-level dependent (BOLD) contrast on a 3.0 Tesla magnetic resonance scanner. We employed a tilted plane acquisition sequence designed to optimize functional sensitivity in the orbitofrontal cortex and medial temporal lobes (Deichmann et al., 2003). To cover the whole brain with a short TR (1.95 s), we used the following parameters: 30 slices, 2 mm slice thickness, 2 mm interslice gap. T1-weighted structural images were also acquired, coregistered with the mean EPI, normalized to a standard T1 template, and averaged across subjects to allow group level anatomical localization. EPI images were analyzed in an event-related manner, within a general linear model, using the statistical parametric mapping software SPM5 (Wellcome Trust center for NeuroImaging, London, UK). The first 5 volumes of each session were discarded to allow for T1 equilibration effects. Preprocessing consisted of spatial realignment, normalization using the same transformation as structural images, and spatial smoothing using a Gaussian kernel with a full-width at half-maximum of 6 mm.

We used two different statistical linear regression models for our analyses. In both every trial was modeled as having two time points, corresponding to cue and outcome onsets. In the first model, two separate regressors were created for cues and outcomes, respectively modulated by the Q values and prediction errors computed by our optimized algorithm. In the second model, 12 separate regressors were created corresponding to the two time points (cues and outcomes) times the two conditioning phases (first and second half of each session) times the three conditions (reward, neutral, and punishment). In all cases, the regressors of interest were convolved with a canonical hemodynamic response function (HRF). To correct for motion artifact, subject-specific realignment parameters were modeled as covariates of no interest. Linear contrasts of regression coefficients were computed at the individual subject level and then taken to a group level random-effects analysis. At group level, we performed two statistical analyses: first a one-sample t test to find brain regions where regression coefficients were significant across subjects, and second a correlation with individual payoffs to find brain regions where regression coefficients increased with higher conditioning effect. A threshold of p < 0.05 after familywise error (FWE) correction for multiple comparisons was applied to avoid any a priori on brain localization. A more liberal threshold (p < 0.001, uncorrected) was also used to observe the extension of significant activations. To further illustrate activations, time courses were estimated by fitting a flexible basis set of finite impulse responses (FIRs), separated from the next by one scan (1.95 s). Both regression coefficients and time courses were then averaged across subjects, pooling together the voxels that passed the conservative threshold in statistical parametric maps (SPMs).

## Supplemental Data

Document S1. One Figure and Supplemental Text
