# Supplementary material for: Subliminal Instrumental Conditioning Demonstrated in the Human Brain
Source: Neuron. 2008 Aug 28;59(4):561–7. doi: 10.1016/j.neuron.2008.07.005 (PMC2572733; doi:10.1016/j.neuron.2008.07.005)
Supplement: Document S1. One Figure and Supplemental Text [file mmc1.pdf]

Neuron, Volume *59*

Supplemental Data

Subliminal Instrumental Conditioning

Demonstrated in the Human Brain

Mathias Pessiglione, Predrag Petrovic, Jean Daunizeau, Stefano Palminteri, Raymond J. Dolan, and Chris D. Frith

Instructions

The aim of the game is to win money, by guessing the outcome of a button press.

At the beginning of each trial you must orient your gaze towards the central cross and pay attention to the masked cue. You will not be able to perceive the cue which is hidden behind the mask.

When the interrogation dot appears you have 3 seconds to make your choice between

- holding the button down
- leaving the button up

If you change your mind you can still release or press the button until the 3 seconds have elapsed.

‘GO!’ will be written in yellow if, at the end of the 3 seconds delay, the button is being pressed.

Then we will display the outcome of your choice. Not pressing the button is safe: you will always get a neutral outcome (£0). Pressing the button is of interest but risky: you can equally win £1, get nil (£0) or lose £1. This depends on which cue was hidden behind the mask.

There is no logical rule to find in this game. If you never press the button, or if you press it every trial, your overall payoff will be nil. To win money you must guess if the ongoing trial is a winning or a losing trial. Your choices should be improved trial after trial by your unconscious emotional reactions. Just follow your gut feelings and you will win, and avoid losing, a lot of pounds!

Figure S1. Computational Modeling

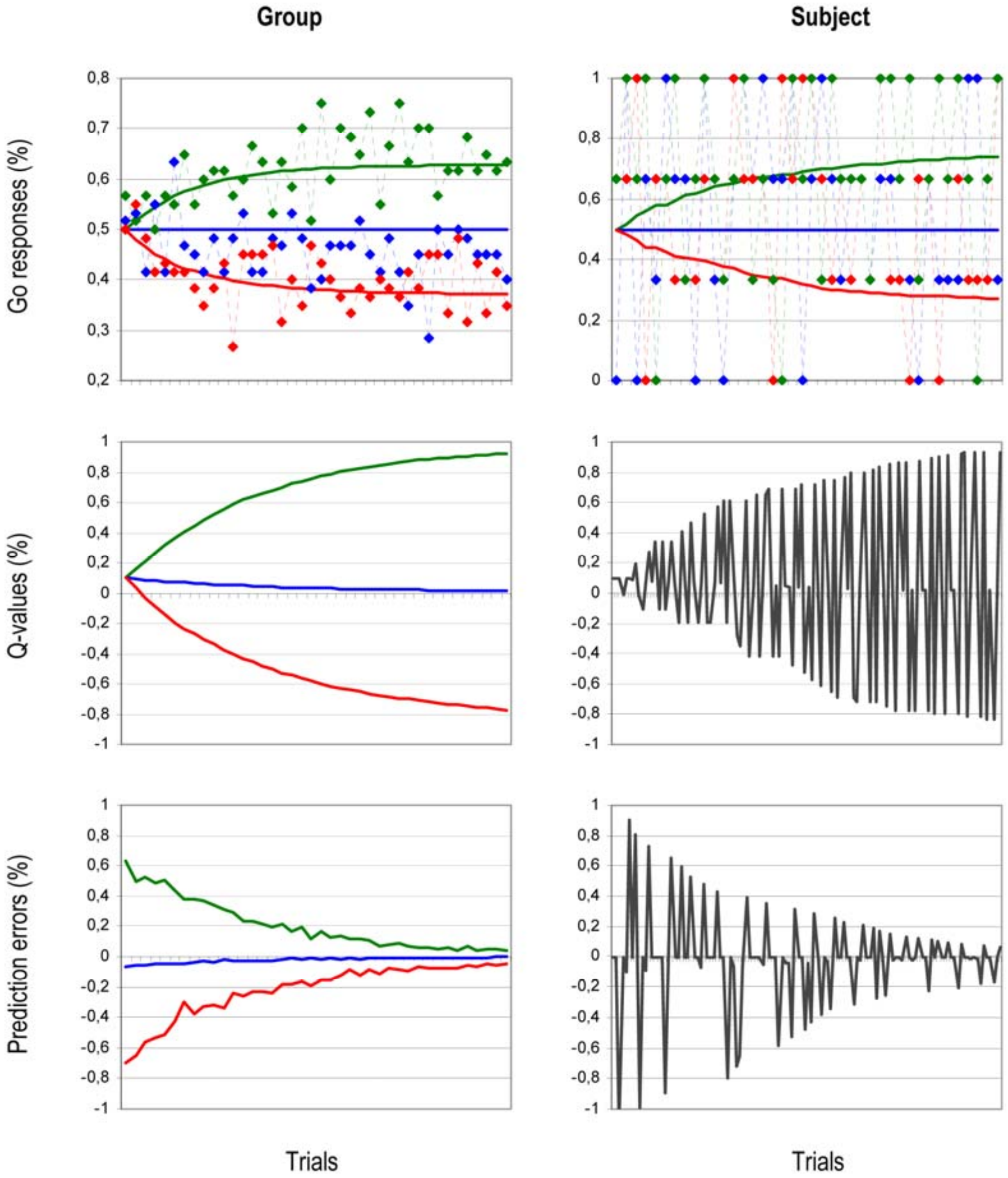

Different measures and outputs are illustrated at group (left) and individual (right) level. The subject chosen is a typical low learner (£4.7 per session on average). Top: Learning curves obtained when button presses are rewarded (green), neutral (blue), or punished (red). Diamonds represent, across trials, percentages of subjects that pressed the button. Continuous lines represent the probabilities of button press estimated by the optimized Q-learning model. Middle and bottom: monetary Q-values and prediction errors generated across trials by the optimized Q-learning model. Colour code corresponding to the different conditions was suppressed at individual level, to better illustrate regressors used for neuroimaging data analysis.
